# Understanding Patient Anxieties in the Social Media Era: Qualitative Analysis and Natural Language Processing of an Online Male Infertility Community

**DOI:** 10.2196/16728

**Published:** 2020-03-10

**Authors:** Vadim Osadchiy, Jesse Nelson Mills, Sriram Venkata Eleswarapu

**Affiliations:** 1 Division of Andrology, Department of Urology David Geffen School of Medicine University of California, Los Angeles, CA United States; 2 Consortium for Health Activity on Social Media David Geffen School of Medicine University of California, Los Angeles, CA United States

**Keywords:** social media, infertility, male, online social networking, Reddit, discussion board

## Abstract

**Background:**

Couples struggling with infertility are increasingly turning to the internet for infertility-related content and to connect with others. Most of the published data on infertility and the internet only address the experiences of women, with limited studies focusing exclusively on internet discussions on male factor infertility.

**Objective:**

The aim of this study was to understand the concerns and experiences of discussants on an online male infertility community and to provide insight into their perceptions of interactions with health care professionals.

**Methods:**

Using the large-scale data analytics tool BigQuery, we extracted all posts in the r/MaleInfertility community (877 members) of the social media website and discussion board Reddit from November 2017 to October 2018. We performed a qualitative thematic analysis and quantitative semantic analysis using Language Inquiry and Word Count 2015 of the extracted posts to identify dominant themes and subthemes of discussions. Descriptive statistics and semantic analytic Z-scores were computed.

**Results:**

From the analysis of 97 posts, notable themes and subthemes emerged: 70 (72%) posts shared personal experiences, including feeling emasculated or isolated or describing a negative (28/97, 29%), positive (13/97, 13%), or neutral (56/97, 58%) experience with a health care professional; 19% (18/97) of the posts posed questions about personal semen analysis results. On the basis of semantic analysis, posts by men had higher authenticity scores (Z=3.44; *P*<.001), suggesting more honest or personal texts, but lower clout scores (Z=4.57; *P*<.001), suggesting a more tentative or anxious style of writing, compared with posts by women.

**Conclusions:**

To our knowledge, this study represents the first evaluation of a social media community focused exclusively on male infertility using mixed methodology. These results suggest a role for physicians on social media to engage with patients and connect them to accurate resources, in addition to opportunities to improve in-office patient education.

## Introduction

### Background

Social media has emerged as a potent resource for patients seeking both anonymous and nonanonymous engagement on acute and chronic medical problems. Despite the ubiquity of social media platforms, a comprehensive scientific understanding of the content of online health-related discussions remains poorly studied, even though 72% of internet users searched for health information on the Web [1]. Reproductive medicine, in particular, has seen a burgeoning presence on the Web, encompassing everything from instructional websites explaining in vitro fertilization [2] to direct-to-consumer marketing of gamete cryopreservation targeting busy career professionals [3].

A majority of couples struggling with infertility turn to the internet for infertility-related content and to connect with others going through similar experiences [4,5]. Despite the high prevalence of male factor infertility [6], a disproportionate number of infertility investigations focus exclusively on women [7]. Similarly, most of the published data on infertility and the internet address only the experience of women [8-11]; only one recently published study [12] focuses exclusively on internet discussions on male factor infertility. Online discussion boards afford discussants with anonymity, allowing for productive conversations related to traditionally private or taboo topics. Published work on online discussion boards of such topics include sexually transmitted diseases [13], suicide [14], and psychosis [15]. Furthermore, discussants do not feel similar pressures as they might during in-person discussions; individuals can share as little or as much as they are comfortable with in this type of forum [16].

Founded in 2005, the discussion website Reddit has become one of the most popular internet destinations in the United States, with more Web traffic devoted to it than other social media websites, including Twitter and Instagram [17]. Reddit users post anonymously on subforums or subreddits related to a specific topic [18]. For example, the male infertility subreddit, r/MaleInfertility, was created “[f]or males with insufficient sperm to achieve pregnancy in fertile females without assisted reproduction and those affected” with the goal of “speak[ing] openly and honestly about our sperm” [19].

### Objectives

The objective of this study was to understand the concerns and experiences of discussants in this online male infertility community, to provide insight into their perceptions of interactions with health care professionals, and to explore differences in the experiences of men and their partners on the Web.

## Methods

### Data Extraction and Processing

We extracted all posts from the Reddit community *MaleInfertility* from November 2017 to October 2018 (12 months). At the time of writing, this open-access online community had 877 members [19]. Posts were extracted using BigQuery (Google LLC, Menlo Park, California), a Structured Query Language–based enterprise data analytics platform, from a dataset uploaded for public use [20]. At the time of data extraction, this time frame represented the most recent 12 months of data uploaded to BigQuery. We retrieved post title, content, author username, and date and time of publication. Posts that were empty or comprised exclusively of the text “[deleted]” or “[removed]” were excluded from analysis.

### Qualitative Thematic Analysis

We performed a qualitative analysis on the extracted data using an inductive, data-driven approach for content analysis of the free-text narrative data, with grounded theory and a constant comparative method as methodology [21,22]. During open coding, one investigator (VO) carefully analyzed text from each post to identify preliminary themes. We defined our unit of analysis as an entire post, given that we were analyzing free-text data without a word limit, one post could therefore contain more than one code. These preliminary themes were then discussed among all authors. On repeat reviews of the data, themes were finalized and then further divided into subthemes for better characterization. Previous studies did not inform initial coding as we used a purely inductive approach. During our review, we also collected data on whether the post was authored by a male or a female partner; this distinction was possible as authors frequently introduced themselves, or this information could easily and reasonably be deduced through language, such as “my husband was told that...” or “my sperm count is....” If there was any ambiguity, we did not assign a gender to the author. As Reddit represents an anonymous social media forum, we were limited to deducing the gender of a participant from the content of his or her post. We also collected data on whether interventions related to male infertility were mentioned.

### Consideration of Researcher Characteristics, Reflexivity, and Mitigation of Biases

The principal evaluators for this qualitative study were adult males; one is a medical student pursuing urological training, and the other two are urologists with advanced fellowship training in male reproductive medicine (andrology). Inherent biases relate to these researchers’ daily interaction with men struggling with infertility, both in the outpatient clinical and outpatient surgical realms. The researchers recognized the potential for bias in selecting themes and identifying representative discussions of male infertility from social media. Themes were discussed and agreed upon in committee by the authors.

### Semantic Analysis

To conduct a semantic-based analysis, we used Language Inquiry and Word Count (LIWC) 2015, an empirically validated textual analysis program capable of translating language into quantitative metrics related to different psychological processes (affective, social, cognitive, perceptual, and others) and linguistic dimensions (parts of speech, grammar, and others) [23]. Previous studies have used LIWC 2015 for similar purposes [24-27]. For our analysis, we used the four summary variables available on LIWC 2015, which were developed and validated using previously published datasets comprising large comparison samples [23,28-31]: (1) analytical thinking, (2) clout, (3) authenticity, and (4) emotional tone. Scores for each of these variables range from 0 to 100. Compared with lower analytical thinking scores, higher analytical thinking scores suggest language that is more formal and logical. Higher clout suggests that the writer is confident and speaks from the perspective of an expert, compared with lower scores that suggest a more tentative or anxious style. Text that scores higher on authenticity reflects a more honest and straightforward style, compared with lower authenticity scores that suggest a less candid and more guarded text. For emotional tone, compared with a lower number, a higher number reflects a more positive tone, with a score of 50 indicating a neutral tone.

We compared means of the aforementioned summary variables between posts authored by men vs women using Mann-Whitney *U* tests. RStudio version 1.1.463 (RStudio, Inc, Boston, Massachusetts) was used for statistical analysis, with *P*<.05 considered statistically significant.

### Ethics

As this study involved anonymous, publicly available data, it was deemed as exempt by the institutional review board of the University of California, Los Angeles. This is consistent with previous investigations of research on social media data [26,32].

## Results

### Overview

A total of 133 posts were initially extracted. A total of 97 posts by 73 unique users remained for analysis after applying exclusion criteria. From these 97 posts, men authored 53 posts (55%) and women authored 21 posts (22%), and gender was not identifiable among 23 posts (24%). The top five most frequently used words were as follows: sperm, DNA, test, motile/motility, and normal. The average word count for all posts was 191 words.

### Sharing Personal Experiences

A total of 72% (70/97) of the posts were related to *Sharing Personal Experiences*. The experiences authors described were often emotionally charged, featuring the subtheme of *Feeling Emasculated or Isolated*, despite frequently mentioning potential sources of support, such as a wife or close partner:

Male infertility is one of the last great taboos. And I can understand why. When my wife and I were struggling to make a baby I found it hard to vocalize my feelings. My failure at fatherhood ate away at my very being and made me feel less of a man.

I also feel like I've failed my wife and that I'm less of a man now. It's depressing. I feel like someone died.

A total of 25% (24/97) of all the posts mention an interaction with a health care professional. A majority (56/97, 58%) of these interactions were neutral; 29% (28/97) of the interactions were negative and 13% (13/97) of the interactions were positive. Negative interactions were often driven by distrust in their health care professional with respect to financial conflicts of interest or an overall distrust in their provider’s fund of knowledge:

At our consultation with the fertility clinic the doctor immediately started pushing IVF, not suggesting any drugs or any other treatments [...] She didn't really have any answers for me and to me seemed to be more focused on female issues than male issues.

His doctor couldn't tell him much, and couldn't explain his morphology results at all. Obviously a great doctor.

Positive experiences related to health care professionals often centered around providing hope and reassurance:

The urologist was pretty encouraging, saying he’s only ever seen 3 people who never regained sperm, but it’s still hard to be positive and not be scared that you’ll be in that small group.

Although, I am crushed, my male infertility urologist (UCLA) to be exact told me that in about 10-20 years (give or take) stem cell technology will be able to help me father a child.

### Searching for Shared Experiences

Complementing the aforementioned theme, 35% (34/97) of the posts involved *Searching for Shared Experiences*. Discussions that included this theme were often discussed within the context of interventions related to infertility, with 35% (34/97) of the posts mentioning such an intervention.

Only a limited subset (5/97, 5%) of posts mentioned intrauterine insemination, but when this topic was featured, it was often related to searching for others who went through the process:

My wife and I have chosen a donor and will start with iui in the next few months [...] What I’ve really felt is missing or that I need is to read about other people’s experiences in this same (or similar) situation. Are there blogs or other resources about this sort of thing? I want to know how other men have dealt with his, how/if they’ve talked to people about it, etc.

Has anyone experienced this? My heart aches for my husband and our future. I cannot imagine going through with a donor sperm at this point. This is such a lonely and isolating experience.

Similar discussions featured in vitro fertilization (mentioned in 12/97, 12% of all the posts) and microdissection testicular sperm extraction (14/97, 14% of all the posts).

My wife and are undergoing IVF but I am so stressed about it not working it's hard to enjoy. I can't talk to anyone about it so I thought I'd try here. Any advice?

We are looking at MICRO TESE to see where to from here, worried that they will not find sperm is what is stressing me at the moment. Reading all the stories I know now that I’m not alone, that is a bit comforting.

### Sharing Resources or Information

A total of 14% (14/97) of the posts were related to *Sharing Resources or Information*. Shared information came in the form of (1) alternative online discussion boards targeted at a group of individuals going through a similar experience, such as using donor sperm, or (2) sharing recently published, peer-reviewed research related to male factor infertility, assisted reproductive technology, and related topics:

We need your help brining a subreddit to life: Parents of Donor Conceived Persons [link]. It's a new subreddit for parents of children conceived using donor sperm, eggs, or embryos [...] This subreddit aims to create a much needed outlet and community. It's a place to share our struggles and solutions in order to raise the best children we can. Please subscribe and share with others.

I have researched this [DNA fragmentation] for a long time and have spent so much time trying to understand all that is involved and I hope you guys find it useful [...] This is a photo representation of what this looks like if you are a visual person [link to figure from peer reviewed article]

### Medication Side Effects

A minority of posts (3/97, 3%) included discussions related to medication side effects, mainly clomiphene. Although 14% (14/97) of all the posts mentioned this medication, it was frequently not within the context of side effects:

Have any guys noticed weight gain while taking Clomid? If so, did it drop back off after stopping it?

### Questions Related to Personal Semen Analysis

A total of 19% (18/97) of the posts featured a question related to a personal semen analysis result. Many authors expressed anxiety or a feeling that they needed to act based on their semen analysis results, especially when the results were available before an appointment with a fertility specialist.

Getting in to see the fertility doctor isnt possible until my Sonohysterogram, and our GP most likely won't know what to make of this [...] Is there anything that can help increase his SA results?

I got my results back, waiting to see the doctor next week but got curious about what this means.

Many questions related to semen analysis results also emerged even after a recent visit to a health care professional to discuss their workup. Similar to the aforementioned subtheme related to negative experiences with health care professionals, authors expressed uncertainty related to the interpretation of their results by health care professionals.

I'd really like some input on these numbers. How low are they, really? Is Doc1 right on her analysis?

my urologist said that this lower morphology will impact fertility but did not provide any statistics... does anyone know the numbers behind how much worse off I am

### Semantic Analysis

A semantic analysis revealed differences in the linguistic attributes of posts authored by men vs their partners (Table 1). Posts authored by men had higher authenticity scores (Z=3.44, *P*<.001), suggesting a more honest or personal text, but lower clout scores (Z=−4.57, *P*<.001), suggesting a more tentative or anxious style of writing, compared with posts by women. No differences emerged in analytical or tone scores.

**Table 1:** Semantic analysis of the linguistic attributes of posts that are authored by men compared to those of their partners.

Variable	All	Men	Women	Z	*P* value
Analytical	56.36	54.18	47.55	1.38	.17
Authentic	44.09	57.74	30.63	3.44	<.001^a^
Clout	51.92	37.21	65.26	−4.57	<.001^a^
Tone	40.72	34.45	46.30	−0.90	.37

^a^Statistically significant.

In Table 1, Mann-Whitney *U* tests for significance were used to outline mean differences of the four summary variables between posts authored by men vs their partners. Note that mean values under the *All* category include data from both men and women, in addition to data from posts where author gender could not be identified.

## Discussion

### Principal Findings

To our knowledge, this study represents the first evaluation of a social media community focused exclusively on male infertility utilizing mixed methodology, with both a classic qualitative analysis and natural language processing methods. For many men and their partners, male factor infertility is stigmatized [33]. Online discussion boards, such as the subreddit we analyzed here, create a space for discussants to connect with others anonymously and to ask questions that they may not feel comfortable sharing in person with their physicians. Results from our analysis may inform strategies for enhanced communication with male infertility patients and their partners, both on the Web and in the clinic.

Many of this study’s findings are consistent with previous studies on online infertility discussions, which describe the struggle of infertility as a profoundly emotional and psychologically trying period in the lives and relationships of discussants [10,34]. Hanna et al [35], in a qualitative analysis of an online infertility forum, underscore that regardless of etiology, infertility represents an “emotional rollercoaster” for both partners, with strong feelings (positive and negative) on both ends of the spectrum. Perhaps attributed to our exploration of an online discussion board focused exclusively on male factor infertility, we found that discussions related to sharing personal experiences were often negative. Subthemes involving feelings of emasculation and isolation permeated through the majority of posts we analyzed, supporting the idea that for at least a subset of men, the ability to conceive a child may be tied to their senses of masculinity and self-worth. It is interesting to note that Beeder et al [12], who also performed a content analysis of a different group of online discussions on male factor infertility, observed a similar theme: *Feelings associated with male infertility*. This theme, however, was featured in only 16% of the posts, compared with 72% of the posts in our analysis. Feelings of inadequacy were identified in less than 1% of the posts in the study by Beeder et al [12]. This discrepancy may be at least partially explained by differences in the proportion of women to men authoring the analyzed posts. Compared with over 60% of the posts authored by women in the study by Beeder et al [12], this study had fewer than 30% of the posts authored by women where gender could be identified. We found that discussants also frequently used this forum to connect with others to address these aforementioned feelings of isolation, thereby creating an opportunity for discussants to normalize each other’s experiences [34-36].

Many of the experiences shared involved interactions with health care providers; nearly 30% of these interactions were negative. Perceived poor physician communication represented the cornerstone of many of these negative experiences. In addition, almost 20% of the posts involved a question related to the interpretation of semen analysis results, even after a recent visit to a health care professional. A similar finding was reported by Beeder et al [12], where they noted that almost all the questions related to male infertility diagnosis and testing were about interpretation of semen analysis results. These findings suggest a potential role for physicians, in the office or on social media, to engage with patients and connect them to accurate resources. This engagement is particularly critical as the accuracy of health information on the Web is circumspect [37]. One study found that even websites of fertility clinics affiliated with the Society for Assisted Reproductive Technology failed to meet most of the American Medical Association’s health information guidelines [38].

Although this forum was focused on male factor infertility, both men and their partners participated in discussions. Quantitative findings from the semantic analysis reveal similarities and differences in the ways that men and women communicate on this forum. Posts by men were characterized by an overall less confident writing style (lower clout scores), compared with those authored by women. These findings are aligned with the results of a previous study by Hanna et al [35], which noted that the language men use on the Web when discussing even highly personal issues related to infertility remains constrained by norms of hegemonic masculinity. The results of our semantic analysis may reflect attitudes and behaviors that occur even outside of online discussion boards. In a study exploring the infertility experience of Polish couples, Nagorska et al [39] found that women were more likely to talk openly and confidently about infertility, whereas men found themselves acting more restrained, consistent with the lower clout scores we observed in this study. Despite these constraints, posts by men were more honest and personal (higher authenticity scores), compared with those of women, perhaps underscoring the value of anonymity on an online discussion board [40]. The finding of lower clout and higher authenticity scores is consistent with the results of our qualitative analysis, as the subtheme of *Feeling emasculated or isolated* featured posts that were authored primarily by men. Online forums may serve a particularly important role for men struggling with infertility, as men are less likely to seek in-person social support to cope with infertility stress [41]. Posts authored by men vs women did not differ in their tone (both were equally negative) or analytical scores (same degree of formality and logical thinking patterns).

Although anonymity represents a valuable benefit to participating in an online forum on infertility, it also creates difficulties in analyzing participant demographics. As only the username was available, we were limited to deducing the gender of a participant from the content of the post; this limits statistical power and perhaps introduces sampling bias to our semantic analysis. In addition, individuals who turn to the internet for health care information may be different with respect to demographics and information preferences from those who do not [42]; the results of this study should therefore be interpreted within this context. To our knowledge, the subreddit we have analyzed represents the largest community on Reddit focused on male factor infertility. Future studies may consider an expanded analysis incorporating other online discussion boards that also focus on male factor infertility.

### Conclusions

Although online discussion boards may serve patients’ needs in a different yet complementary way to their experiences with health care providers in person, this study underscored a need to enhance in-office communication, especially within the context of male factor infertility. The semantic analysis suggests that the online and in-office needs of men and their partners differ, especially with respect to infertility that is male factor in etiology. This study’s results also suggest a potential role for physicians on social media to engage with patients and connect them to accurate resources.

## Abbreviations

LIWC: Language Inquiry and Word Count
